# Extreme Obesity (BMI ≥ 60 kg/m^2^) Characterization up to and Beyond 80 kg/m^2^ in 1,262,454 Bariatric Surgery Patients

**DOI:** 10.1002/oby.70289

**Published:** 2026-08-24

**Authors:** Vance L. Albaugh, Hector Garcia Navas, Robert C. Ross, Florina Corpodean, Michael Kachmar, Denise M. Danos, Steven B. Heymsfield, Peter Katzmarzyk, Michael W. Cook, Philip R. Schauer

**Affiliations:** 1 Metamor Institute, Pennington Biomedical Research Center, Baton Rouge, Louisiana, USA; 2 Department of Surgery, Louisiana State University Health Sciences Center, New Orleans, Louisiana, USA; 3 Franciscan Missionaries of Our Lady Health, Baton Rouge, Louisiana, USA; 4 Department of Behavioral & Community Health, Louisiana State University School of Public Health, New Orleans, Louisiana, USA; 5 University Medical Center, New Orleans, Louisiana, USA

**Keywords:** bariatric surgery, extreme obesity, high BMI, severe obesity

## Abstract

**Objective:**

This study aimed to estimate extreme obesity prevalence and association with disease burden and outcomes in patients undergoing metabolic and bariatric surgery (MBS).

**Methods:**

Cross-sectional data from the Metabolic and Bariatric Surgery Accreditation and Quality Improvement Program (MBSAQIP, 2015–2023) were analyzed (*n* = 1,795,127). Preoperative disease burden and 30-day outcomes were compared across BMI categories (< 40, 40.0–49.9, 50.0–59.9, 60.0–69.9, 70.0–79.9, and ≥ 80 kg/m^2^), emphasizing extreme obesity compared to lower BMI categories.

**Results:**

Extreme obesity represented 5.3% of the eligible sample, though even higher BMI values (i.e., ≥ 70 kg/m^2^) were consistently present in 1.1% of cases annually (13,677 cases/9 years). Relative to BMI < 40 kg/m^2^, patients with the highest BMI values (≥ 70 kg/m^2^) had significantly greater frequency of hypertension (56.7% vs. 46.9%), sleep apnea (59.6% vs. 34.6%), COPD (2.6% vs.1.1%), and functional dependence (4.5% vs. 0.5%). Increasing BMI category was associated with increased serious complications (2.6% vs. 0.7%; *p* < 0.001) and mortality (0.35% vs. 0.05%; *p* < 0.001).

**Conclusions:**

Extreme obesity is common in MBS, and greater BMI is associated with progressively higher comorbidity burden and complications. Despite the higher frequency of complications, absolute events are low and do not contraindicate multidisciplinary obesity treatment. Strategies to reduce preoperative BMI among patients with the highest BMI values warrant evaluation.

## 1 | Introduction

Severe obesity is a significant driver of cardiometabolic disease, renal disease, and cancer in the United States [1, 2], with higher body mass index (BMI) obesity growing at unprecedented rates in adults [3] and children [4]. Given the changing landscape of obesity, the current standardized clinical classification for obesity diagnosis continues to grow increasingly limited (i.e., Class 1 BMI 30.0–34.9, Class 2 BMI 35.0–39.9, Class 3 BMI ≥40.0kg/m^2^), especially as the fastest-growing BMI cohorts [3] in the United States are those greater than BMI 50 kg/m^2^.

Regardless of the changing obesity landscape, significant bias persists in both the literature and clinical setting with respect to obesity severity and individuals with higher BMI values. Numerous prior studies treat individuals with obesity as a homogeneous category (i.e., BMI ≥ 30 kg/m^2^) or combine wide BMI ranges for analysis (i.e., BMI ≥ 40 kg/m^2^) that may dilute risk gradients and phenotypic differences. Even more concerning, however, is the practice of routinely excluding individuals above certain BMI or body weight thresholds [5–8], suggesting that higher BMI (e.g., BMI ≥ 50 kg/m^2^) is rare or even a data entry error that has been termed “biologically implausible.” This literature bias is accompanied by clinical care bias as well, as primary care and medical subspecialty clinics may not be able to accommodate individuals with increasingly severe obesity [9, 10]. This bias stems not only from building accessibility and clinic equipment with weight limitations (e.g., exam tables), but also diagnostic equipment with low weight maximums or body habitus constraints [9, 10]. Thus, a greater focus on clinical care for higher BMI obesity is needed.

While higher BMI or “extreme obesity” is commonly described in the bariatric surgery literature, there is no formally accepted nomenclature or appreciation of its clinical implications. BMI ≥ 60 kg/m^2^ is a generally accepted threshold associated with increased surgical challenges but, more importantly, when medical optimization and preoperative weight loss are critical for treatment success. Unfortunately, few clinical studies focus on defined groups with higher BMI obesity in the medical literature. In fact, even pharmaceutical clinical trials for obesity medications rarely focus on endpoints for individuals with BMI ≥ 40 kg/m^2^, which is particularly troublesome [11, 12]. Thus, the current study aimed to quantify and characterize individuals presenting for metabolic and bariatric surgery (MBS) to examine the relationship of higher BMI on disease burden and surgical outcomes in patients with extreme obesity (BMI ≥ 60 kg/m^2^). To date, this is the largest sample and the first to describe operative outcomes in patients with BMI ≥ 80 kg/m^2^. We hypothesized that individuals with the highest BMI values would carry the greatest comorbid disease burden and, with the growth in higher BMI categories nationally [3], the proportion of higher BMI cases would increase over time.

## 2 | Methods

### 2.1 | Study Cohort

The current study utilized the Metabolic and Bariatric Surgery Accreditation and Quality Improvement Program (MBSAQIP) dataset [13], the largest clinical database of patients with severe obesity that includes direct anthropomorphic measurements (i.e., directly measured weights obtained with a high-capacity scale). MBSAQIP oversees national accreditation for MBS facilities, given the unique clinical challenges of obesity. A major strength of MBSAQIP is that program standards require facilities, furniture, and equipment that accommodate individuals with obesity, including high-capacity scales for accurate body weight measurements [14]. No other national datasets include similar requirements, allowing confirmation and accurate weight measurements.

MBSAQIP captures preoperative patient characteristics, comorbid disease burden, and surgical case characteristics, as well as 30-day postoperative outcomes. Data are collected by independent clinical data reviewers for all MBS cases performed at MBSAQIP-accredited centers. Deidentified Participant Use Files (PUF) are issued annually, and the current study examined all available patients from 2015 through 2023.

### 2.2 | Variable Selection and Definitions

**FIGURE 1 |:**
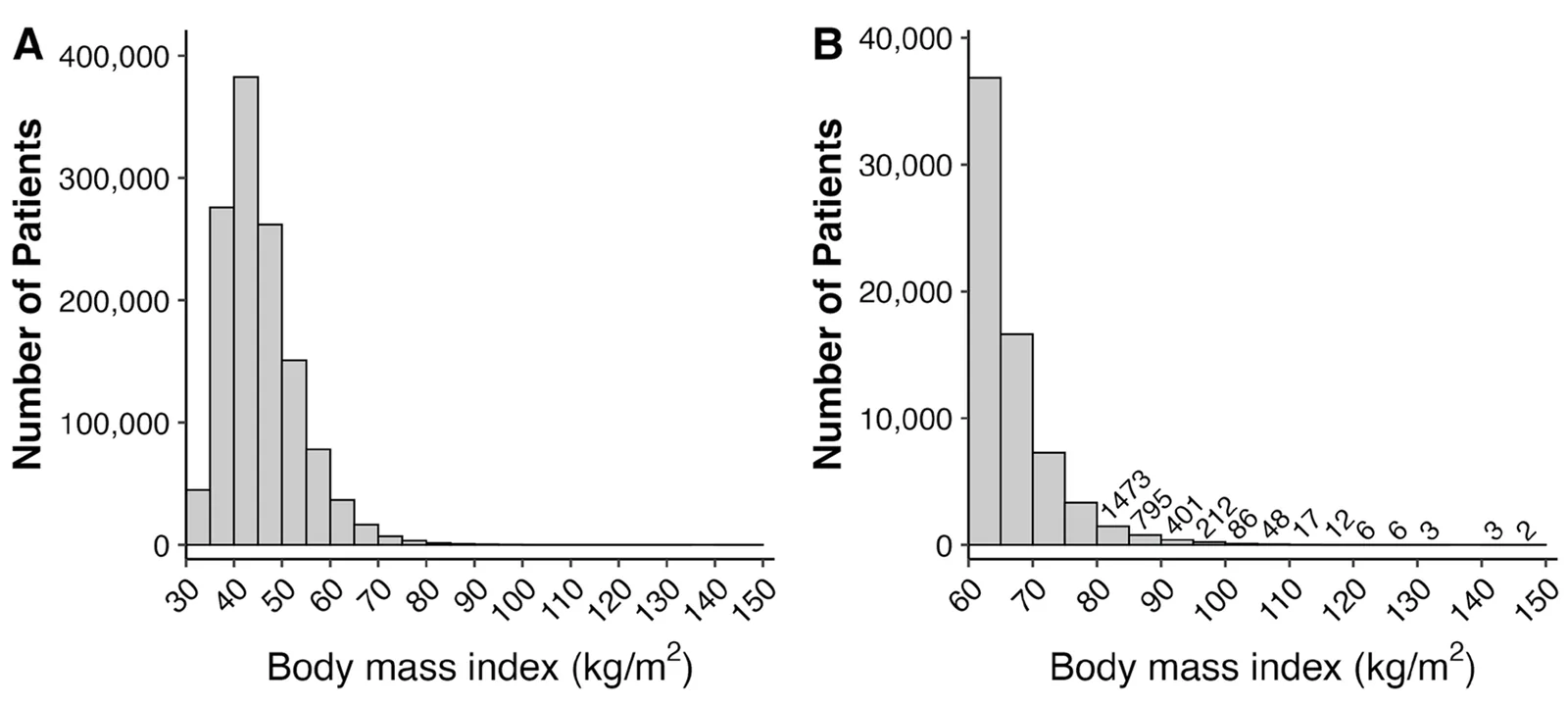
Distribution of preoperative BMI of patients undergoing bariatric surgery over time, MBSAQIP 2015–2023. Primary patient case numbers are shown for (A) BMI from 30 to 150 kg/m^2^ and (B) limited to extreme obesity (BMI ≥ 60 kg/m^2^), with numbers above bars indicating the total cases for that bar for cases with BMI ≥ 80 kg/m^2^.

Given there is no standardized nomenclature for the wide range of obesity severity, the cohort was analyzed categorically using BMI increments of 10 kg/m^2^, with the lowest and highest categories being BMI < 40 and BMI ≥ 80, respectively, based on empirical case distribution (Figure 1). BMI ≥ 60 kg/m^2^ was selected as a primary focus, given that this BMI value indicates approximately 200 lb (91 kg) of extra body weight and is considered an inflection for serious complications and mortality [3]. Moreover, from a surgical perspective, this is the point at which preoperative weight reduction and medical optimization are necessary. Surgical cases were identified using current procedural terminology codes (CPT) for sleeve gastrectomy (43775), Roux-en-Y gastric bypass (43,644; 43,645; 43,846; 43,847), or other bariatric procedures (43,659; 43,845; 43,770; 43,999; 43,843; 43,842). Only primary or “initial” procedures were included in the analysis, as conversion, revision, and emergency cases were excluded.

Analyzed demographic information included the following: sex, age, race, Hispanic ethnicity, and BMI at the time of surgery. Preoperative comorbidities included: history of venous thrombosis requiring therapy, venous stasis, therapeutic anticoagulation, steroid or immunosuppressant use for chronic conditions, pulmonary embolism (PE), diabetes mellitus, chronic obstructive pulmonary disease (COPD), obstructive sleep apnea, gastroesophageal reflux disease (GERD), history of myocardial infarction, history of percutaneous coronary intervention, prior cardiac surgery, hypertension, hyperlipidemia, renal insufficiency, and dialysis. American Society of Anesthesiologists (ASA) class, functional health status, and smoking status within 1 year were also evaluated. Cases with age < 18 years or missing or unknown age, BMI, or surgical approach were excluded.

Perioperative case data included endpoints within 30 days post-operatively that are routinely collected as part of MBSAQIP. These variables included composite infection and serious complications variables. A three-composite major adverse cardiovascular event variable (MACE) and death within 30 days were also collected (see definitions in Table S1).

### 2.3 | Statistical Analysis

Categorical variables were summarized with frequency and percentage, while continuous variables were summarized with mean and standard deviation (SD). Bivariate relationships between BMI and case characteristics were assessed using chi-square tests, while association between age and BMI was assessed using ANOVA. Linear trends in the prevalence of baseline comorbidities and postoperative complications by BMI were assessed with Cochran-Armitage test for trend. Due to limited sample size in the highest BMI category (BMI ≥ 80 kg/m^2^), estimates were underpowered and potentially unstable; we therefore analyzed BMI categorically with the highest category being ≥ 70.0 kg/m^2^. Statistical analyses and/or data visualization were performed in SAS/STAT software, version 9.4 (SAS Institute Inc.) or R Statistical Software (v4.5). All statistical tests were two-sided, with a *p* value < 0.05 used to determine statistical significance.

## 3 | Results

### 3.1 | Case Characteristics and Patient Demographics

From 2015 to 2023 there were 1,795,127 total cases, of which 1,262,454 were analyzed. Total cases increased over time except for 2020 (Table 1), with post-2020 COVID pandemic years having significantly higher case numbers than pre-2020. Extreme obesity (BMI ≥ 60 kg/m^2^) accounted for 5.3% of cases (*n* = 53,464), with a significant number of individuals at higher BMI strata (Figure 1). Annual cases in patients with BMI ≥ 70 accounted for ~1% of total cases on average. This proportion has remained relatively constant, except for the first year of data collection, in which 1.6% of total cases had BMI ≥ 70. While there were a significant number of patients with BMI ≥ 80 (*n* = 3065), these cases represented a low proportion of the overall sample (0.24%). Thus, these cases were categorized descriptively, but interpretation and statistical analysis for the highest BMI group was limited to BMI ≥ 70.

Across BMI categories, case percentages remained relatively unchanged over time (Table 1). While most patients (~50%) had BMI between 40.0 and 49.9, approximately a quarter remained in those with BMI < 40.0. The other quarter of MBS cases were in patients with higher BMI (i.e., BMI ≥ 50). Over time the percentage of cases with BMI 50.0–59.9 remained stable and ranged from 17.8% to 18.9% annually. In stark contrast to US national growth [3], the proportion of higher BMI cases remained unchanged at 4.4% and 1.1% in the BMI 60.0–69.0 and BMI ≥ 70 groups, respectively.

As a percentage of primary MBS cases overall, sleeve gastrectomy was the predominant procedure (68.9%), with Roux-en-Y gastric bypass (RYGB) in a distant second (27.3%), demonstrating that 96.2% of all primary cases were either sleeve or RYGB. However, over time the percentage of sleeve gastrectomy tended to decrease with RYGB increasing slightly (Table 1). With respect to BMI category, BMI ≥ 70 had the lowest percentage of RYGB (23.9%) for all BMI categories, though a similar percentage of sleeve gastrectomy in the BMI ≥ 70 group was observed compared to the overall cohort (67.8% vs. 68.9%). BMI ≥ 70 also had the highest proportion of “other” bariatric procedures, which includes several procedures that have not had standardized data collection across all years, including biliopancreatic diversion with duodenal switch, single anastomosis duodenoileostomy, one-anastomosis gastric bypass, and adjustable gastric banding.

In terms of demographics (Table 2), female sex represented most cases across all BMI groups (80.1%). As BMI increased, however, there was a slight increase in the case proportions of males. With respect to race, non-Hispanic White individuals accounted for the most cases, though non-Hispanic Black individuals accounted for a significantly increasing percentages of overall cases as BMI increased. In contrast, Hispanic individuals accounted for a decreasing percentage of cases in higher BMI categories. Average age decreased with increasing BMI category. In comparison to cases at lower BMI categories, patients with BMI ≥ 70 were younger and tended to have slightly higher percentages of patients identifying as Non-Hispanic Black race.

### 3.2 | Obesity-Associated Comorbidities by BMI Category

Overall, the most common comorbidities were hypertension (45.5%), sleep apnea (38.1%), GERD (29%), diabetes mellitus (25.3%), and hyperlipidemia (23.0%). All remaining comorbidities were present in 7.5% of cases or less. With respect to functional status, 99.2% of individuals were functionally independent, with only 0.6% and 0.2% of cases being either partially or totally dependent, respectively (Table 3).

**FIGURE 2 |:**
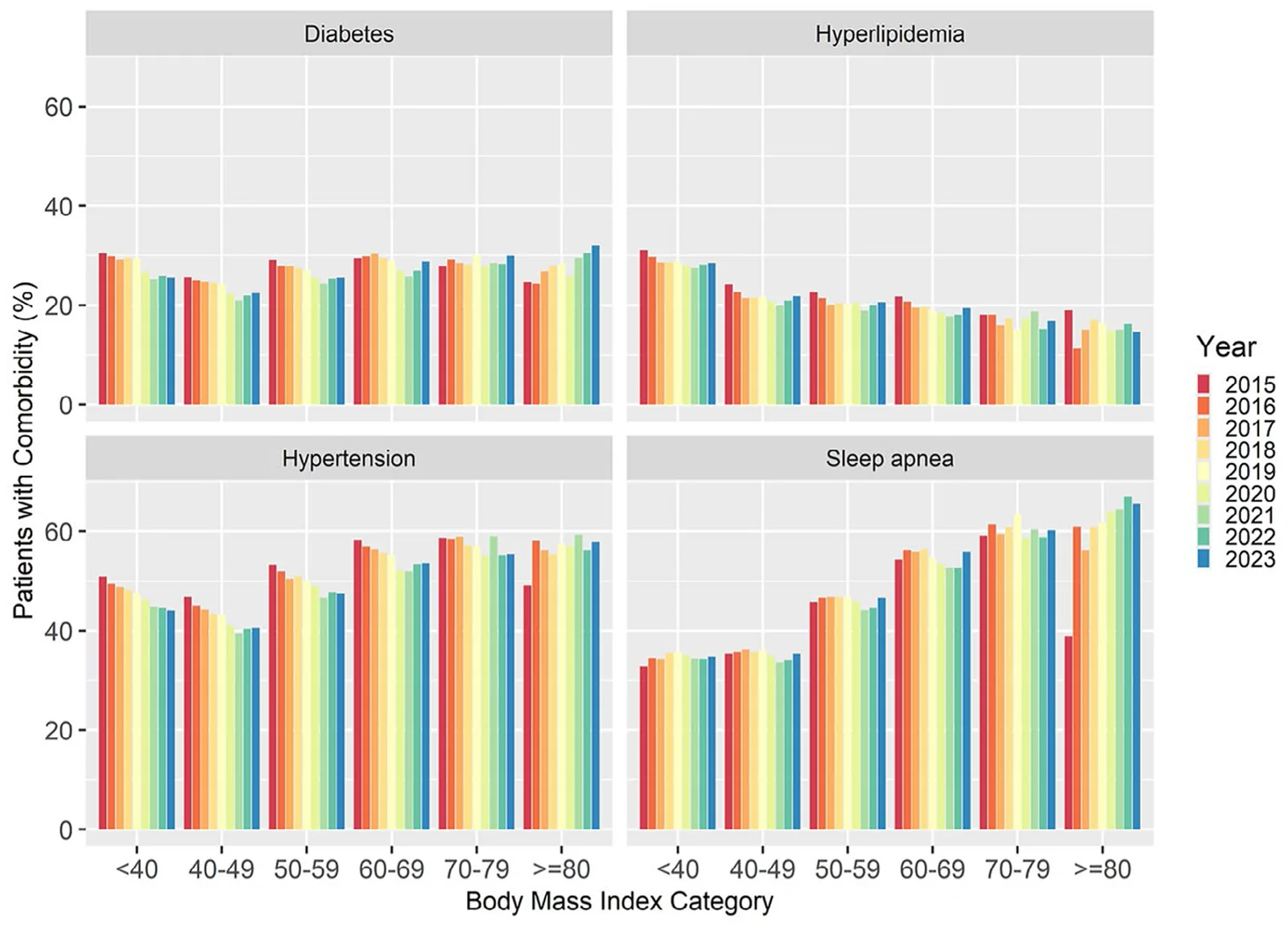
Comorbidity burden of MBS patients over time, 2015–2023. Percentages of bariatric surgery patients with diabetes, hyperlipidemia, hypertension, and sleep apnea within discrete BMI categories by year. Vertical bars indicate year of operation as defined in the legend by color gradient. [Color figure can be viewed at wileyonlinelibrary.com]

When examining case characteristics across BMI categories, there were several notable differences, though all comparisons were significantly different given the large sample (Table 3). While diabetes mellitus was present in 25.3% of patients overall, the BMI 40–49.9 group had the largest proportion of patients without diabetes (76.7%). Non-insulin dependent diabetes was highest in the BMI ≥ 70 group (21.1%), and insulin dependent diabetes was highest in the BMI < 40 group (8.8%). Hypertension, sleep apnea, COPD, history of smoking, therapeutic anticoagulation, history of deep vein thrombosis (DVT)/pulmonary embolism (PE), and venous stasis were most common in patients with BMI ≥ 70. These obesity-associated comorbidities demonstrated BMI-dependent relationships, with trends for gradual increases observed with increasing BMI category, suggesting that increased adiposity is associated with greater disease burden. Over time in categories with BMI < 70, diabetes, hyperlipidemia, and hypertension have trended downward slightly; however, the prevalence of these comorbidities has remained steady or increased (e.g., sleep apnea) in patients with BMI ≥ 70 (Figure 2).

Not all obesity-associated comorbidities (Table 3) showed increasing prevalence with increasing BMI, and several comorbidities were most prominent in the lowest BMI category (BMI < 40). These included GERD (32.0%), hyperlipidemia (28.7%), immunosuppressive therapy (2.2%), previous percutaneous coronary intervention (1.8%), prior myocardial infarction (1.3%), and previous cardiac surgery (1.1%). In fact, BMI ≥ 70 was associated with the lowest likelihood of hyperlipidemia (16.7%), GERD (26.4%), immunosuppressive therapy (1.8%), myocardial infarction (0.8%), and history of percutaneous coronary intervention (0.9%) or cardiac surgery (0.4%).

Regarding functional status, as noted earlier, 99.2% of patients were functionally independent, which is not surprising for an elective surgical dataset. There was a relationship with BMI; however, functional independence gradually decreased as BMI category increased. Even in patients with BMI ≥ 70, the over-whelming majority remained functionally independent (95.1%). Regardless, the BMI ≥ 70 group had the highest proportions of patients who were partially and totally dependent with 3.8% and 0.7%, respectively, with a clear trend of the BMI ≥ 80 group having increased partial or total functional dependence (Table 3).

### 3.3 | Thirty-Day Postoperative Complications

Postoperative complications (Table 4) were relatively low for all BMI categories, though there was a trend for a gradual increase in complication burden with increasing BMI for most endpoints. Proportions of either individual or composite complications were all < 6% overall, though the large sample size led to many small but significant differences between BMI categories. Postoperative transfusion ranged from 0.61% to 0.74% and reoperation ranged from 1.03% to 1.21%, with both being similar across BMI groups and no clear BMI-dependent association.

**FIGURE 3 |:**
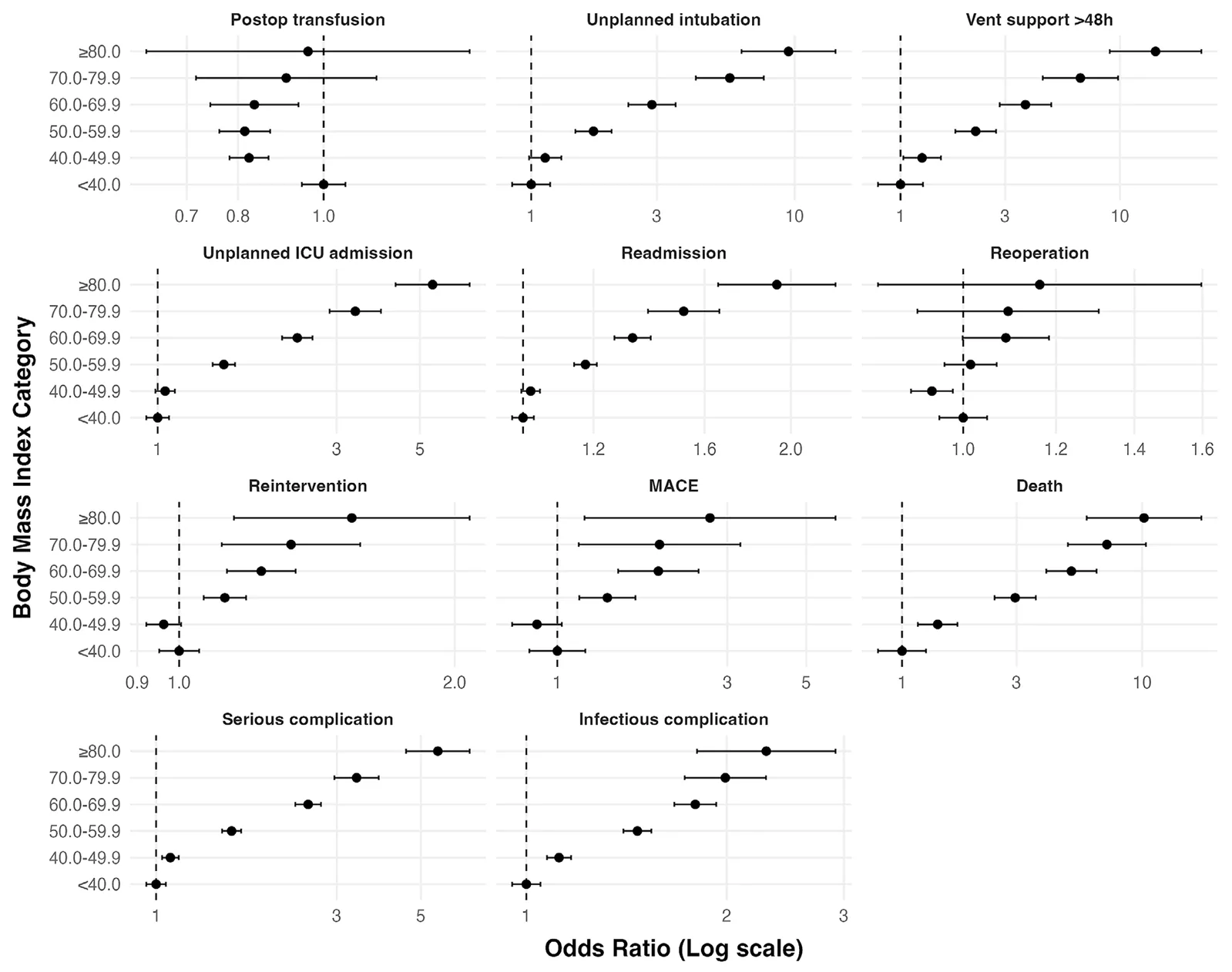
Odds ratios for the association of 30-day postoperative complications and BMI category. Odds ratios for postoperative complications were examined for individual BMI categories (< 40.0, 40.0–49.9, 50.0–59.9, 60.0–69.9, 70.0–79.9, and ≥ 80.0 kg/m^2^), with BMI < 40 kg/m^2^ serving as the reference. Circles represent unadjusted odds ratios and 95% confidence intervals. ICU, intensive care unit; MACE, major adverse cardiovascular endpoint; Postop, postoperative; Vent, ventilatory.

In contrast, the highest BMI (≥ 70 kg/m^2^) group had the highest proportions of most complications, including unplanned intubations (0.56%, *n* = 77), postoperative ventilator requirements (0.36%, *n* = 49), unplanned ICU admission (1.86%, *n* = 254), readmissions (4.94%, *n* = 676), reintervention (1.21%, *n* = 166), MACE (0.15%, *n* = 21), and mortality (0.35%, *n* = 48), as well as composites for serious (2.57%, *n* = 352) and infectious complications (2.05%, *n* = 281). Despite the increased comorbidity burden in the BMI ≥ 70 group, mortality remained low (0.35%, *n* = 48) but was 7 times greater compared to the lowest BMI group < 40 (0.05%, *n* = 145). While underpowered, cases with BMI ≥ 80 compared to a separate BMI 70.0–79.9 category had greater numbers for every complication (Table 4). Unadjusted odds ratios corresponding to each of the quantified 30-day complications (Figure 3) showed a tendency for increasing BMI to be associated with higher odds for unplanned intubation, unplanned ICU admission, ventilatory support for greater than 48 h, readmission, death, serious complications, and infectious complications.

## 4 | Discussion

Disproportionate growth in high BMI populations emphasizes the need to understand the spectrum of obesity severity, especially with respect to prevalence and treatment. MBSAQIP is the largest available dataset of direct anthropometric measurements from a wide range of patients with obesity, allowing for assessment of obesity severity using BMI as an adiposity surrogate [15]. In the current study, 25% of total cases were performed in patients from the fastest-growing BMI categories (i.e., BMI ≥ 50.0) [3]. While BMI ≥ 70 was associated with the highest comorbidity burden, not all comorbidities were positively associated with higher BMI. Despite high BMI obesity growth in the United States, these population trends have not been associated with a meaningful increase in surgical cases in this dataset. Individuals with BMI ≥ 70 continue to make up only ~1% of MBS operations annually despite having low surgical morbidity and mortality. Overall, these findings and others [1, 3, 4] demonstrate that higher BMI values are common and, given their increased complexity, these individual would benefit from combined multidisciplinary approaches to prevent and treat high BMI obesity.

The worsening of adult and pediatric obesity requires a greater understanding of obesity’s effect on cardiometabolic health and a more inclusive obesity nomenclature. The current obesity classification (e.g., Class 1, 2, 3) has limited utility for patients with high BMI [3, 4]. For example, an individual with BMI of 40 kg/m^2^ is not likely similar physiologically to someone with BMI of 60 kg/m^2^, even though both have “Class 3 obesity.” In this study comorbidity burden increased with higher BMI for diabetes, hypertension, and obstructive sleep apnea, consistent with studies in children [16] and adults [1]. In contrast, other comorbidities were unexpectedly overrepresented in lower BMI categories (i.e., BMI < 40), including GERD, hyperlipidemia, prior cardiac surgery, cardiac stenting, and a history of myocardial infarction. While a dose-dependent increase in cardiometabolic and renal disease is observed with increased BMI [1], that relationship was not observed for all comorbidities in the current study. As a surgical quality dataset, however, MBSAQIP represents nonrandomized treatment and is not intended to be population reflective. One potential explanation for enrichment of cardiovascular disease in lower BMI categories is that these patients are undergoing MBS to treat and prevent known cardiovascular disease or risk factors (e.g., diabetes). However, ease of diagnosis (e.g., hypertension vs. dyslipidemia) and comorbidity documentation to meet insurance payer requirements may also contribute to these differences. Regardless, prospective studies are needed to better understand the association between BMI and cardiometabolic disease.

Even though the association between BMI and comorbidity burden is not uniform, this study offers several other critical observations. First, higher BMI individuals do indeed exist, a fact that was previously questioned with higher BMI inaccurately being referred to as “biologically implausible” [17–19]. Second, not only do these values exist, but they are quite common. Individuals with higher BMI (≥ 60 kg/m^2^) contribute to a steady proportion of MBS cases (> 5%) annually, despite continued growth in the population with BMI ≥ 60 that approaches 1 million in the United States [3]. Significant selection bias is present in the current study, however, since only surgical patients are included in MBSAQIP. Patients considered risk prohibitive for surgery are absent, as are those who never obtained surgical consultation for numerous reasons (e.g., lack of insurance coverage, provider/healthcare facility bias [9]). Similarly, bedbound or housebound individuals with immobility are also underrepresented, especially since the study cohort included > 99% of functionally independent patients. If fact, it is quite possible that individuals with the highest BMI values in the current study could represent the “healthiest” individuals with extreme obesity who could also have a socioeconomic advantage as well. Thus, overall these numbers of patients with higher BMI in the current study are likely a fraction of these individuals nationally.

Irrespective of bias, this cohort demonstrates a wide range of clinical obesity severity with varying comorbidity burden, consistent with the concept of obesity subtypes that requires further study. Body weight is a highly regulated, complex phenotype and one that genome wide association studies have shown limited value for identifying individual genetic drivers of obesity [20]. Studies combining genetics and other phenotyping methods, however, have described several putative subtypes [21–25] that not only differ in etiology but also treatment response [21]. It is tempting to speculate on genetic or environmental predisposition driving obesity in these patients with the highest BMI, but future studies must focus on these mechanisms. With the increased availability of highly effective pharmacological tools, identifying the most appropriate and effective multidisciplinary treatments is increasingly important for high-quality care.

Obesity severity is a major driver of cardiometabolic disease risk [1], making obesity treatment critical. As would be expected with any disease, patients presenting with more severe disease (i.e., higher BMI obesity) have a higher likelihood of complications. While patients with BMI ≥ 70 had some of the highest comorbidity burden and rates of postoperative complications, mortality remained low (0.35%) and not risk prohibitive, which echoed findings from a smaller data subset [26]. In fact, observed complication and mortality rates are comparable to, and in some cases lower than, those of other commonly performed operations [26, 27] (e.g., minimally invasive colectomy, knee/hip arthroplasty). Significant selection bias is present in the sample, though, as only surgical patients are represented in the dataset. Thus, it is possible that serious complications and mortality were low because of more complex procedures being done at high-volume centers with adequate resources and multidisciplinary care. Regardless, healthcare providers and patients alike must remain cognizant of the marked intrinsic risk of living with obesity [28]; that is, continuing to live with severe obesity has a much greater long-term risk than the risk of surgical treatment.

This study has numerous strengths, most notably the highest number of patients with confirmed high BMI in any available dataset. Compared to the National Health and Nutrition Examination Survey (NHANES, 2002–2023), the current cohort has a 440-fold greater number of patients with BMI ≥ 60 kg/m^2^, the highest BMI classification recently reported [3]. MBSAQIP is the only national dataset requiring scales to accommodate large individuals, unlike other population surveys. In the case of NHANES, individuals exceeding scale limits are asked to stand with feet on two separate scales and the numbers are added together to calculate weight—a nonstandard technique [29]. While nonstandard measures are better than none, better data collection practices are critically needed.

Study limitations include the cross-sectional design that precludes causal inference, as well as the retrospective nature, potential for data entry errors, and lack of longer-term follow-up > 30 days postoperatively. As noted earlier, the most significant limitation is selection bias, since MBSAQIP includes only surgical patients. Thus, these patients with the highest BMI potentially represent the healthiest high BMI individuals, consistent with BMI ≥ 80 having a slight decrease in comorbidity burden (Table 3). MBSAQIP also reflects treatment based on patient and treatment selection that changes over time; that is, surgical patient selection as well as operation selection by patients that is affected by insurance coverage and other factors. Also, preoperative weight loss—known to have beneficial effects for individuals with extreme obesity—was not examined in the current study due to complexity, but that analysis has been recently published, demonstrating the associated benefits on perioperative outcomes using this same cohort [30]. Finally, while uniformly using BMI to diagnose obesity is limited [31], it remains a highly accurate surrogate for excess adiposity [15].

## 5 | Conclusion

High BMI and extreme obesity are common and associated with a higher comorbid disease burden that requires multidisciplinary care. Even though MBS is safe and feasible for high BMI obesity, it remains grossly underutilized. While multidisciplinary care is needed to address obesity treatment and prevention, diverse scientific approaches must focus on the phenotypic differences in these individuals with high BMI who have been historically understudied.

## Supplementary Material

Supplementary Information

Additional supporting information can be found online in the Supporting Information section. Table S1**:** Perioperative outcome definitions.

## Figures and Tables

**TABLE 1 |:** Preoperative BMI of bariatric surgery patients over time, MBSAQIP 2015–2023.

	2015	2016	2017	2018	2019	2020	2021	2022	2023
All, *n*	100.0 (111,172)	100.0 (124,392)	100.0 (134,703)	100.0 (138,336)	100.0 (138,846)	100.0 (123,785)	100.0 (155,958)	100.0 (173,237)	100.0 (162,025)
BMI, % (*n*)									
Less than 40	24.3 (27,036)	25.1 (31,180)	25.4 (34,276)	25.6 (35,393)	25.5 (35,375)	25.4 (31,502)	25.4 (39,654)	26.0 (45,084)	26.0 (42,152)
40–49	50.7 (56,344)	51.0 (63,416)	51.1 (68,884)	51.2 (70,801)	51.4 (71,334)	51.3 (63,532)	51.4 (80,154)	51.0 (88,413)	50.5 (81,881)
50–59	18.9 (20,982)	18.5 (23,015)	18.1 (24,355)	18.0 (24,963)	17.9 (24,876)	18.0 (22,317)	18.1 (28,205)	17.8 (30,890)	18.1 (29,299)
60–69	4.5 (5026)	4.4 (5467)	4.3 (5745)	4.2 (5800)	4.1 (5758)	4.2 (5192)	4.1 (6400)	4.1 (7131)	4.3 (6945)
70–79	1.0 (1093)	0.8 (1022)	0.8 (1144)	0.8 (1090)	0.9 (1186)	0.8 (1014)	0.8 (1233)	0.8 (1404)	0.9 (1426)
80 or greater	0.6 (691)	0.2 (292)	0.2 (299)	0.2 (289)	0.2 (317)	0.2 (228)	0.2 (312)	0.2 (315)	0.2 (322)
**Sleeve gastrectomy**									
All, *n*	71,826	84,645	94,754	98,476	98,136	85,966	107,929	118,581	110,132
% Annual cases	64.6	68.0	70.3	71.2	70.7	69.4	69.2	68.5	68.0
BMI, % (*n*)									
Less than 40	25.4 (18,240)	25.8 (21,825)	26.2 (24,805)	26.3 (25,909)	26.2 (25,687)	26.4 (22,681)	26.5 (28,588)	27.1 (32,082)	27.2 (29,960)
40–49	51.4 (36,891)	51.9 (43,926)	51.7 (49,026)	51.8 (51,020)	51.9 (50,931)	51.6 (44,351)	51.8 (55,904)	51.5 (61,017)	50.9 (56,092)
50–59	17.5 (12,592)	17.3 (14,639)	17.1 (16,159)	16.9 (16,601)	16.9 (16,561)	17.0 (14,640)	16.8 (18,147)	16.5 (19,598)	16.8 (18,471)
60–69	4.2 (2991)	4.0 (3398)	4.0 (3768)	4.0 (3979)	4.0 (3916)	4.0 (3411)	3.9 (4216)	4.0 (4714)	4.0 (4435)
70–79	0.9 (646)	0.8 (652)	0.8 (787)	0.8 (760)	0.8 (810)	0.8 (710)	0.8 (843)	0.8 (952)	0.9 (951)
80 or greater	0.6 (466)	0.2 (205)	0.2 (209)	0.2 (207)	0.2 (231)	0.2 (173)	0.2 (231)	0.2 (218)	0.2 (223)
**Roux-en-Y gastric bypass**									
All, *n*	34,701	34,660	35,444	35,530	36,375	33,418	41,849	47,538	45,120
% Annual cases	31.2	27.9	26.3	25.7	26.2	27.0	26.8	27.4	27.8
BMI, % (*n*)									
Less than 40	21.2 (7349)	21.5 (7449)	22.1 (7841)	22.8 (8115)	23.1 (8414)	22.9 (7668)	23.1 (9661)	24.0 (11,396)	24.3 (10,959)
40–49	50.5 (17,522)	50.7 (17,575)	51.6 (18,286)	51.4 (18,255)	51.7 (18,820)	52.3 (17,462)	51.9 (21,703)	51.5 (24,501)	51.1 (23,062)
50–59	21.6 (7505)	21.5 (7450)	20.6 (7292)	20.7 (7337)	20.0 (7280)	19.7 (6589)	20.2 (8474)	19.8 (9411)	19.7 (8869)
60–69	5.1 (1767)	5.2 (1794)	4.7 (1661)	4.2 (1498)	4.2 (1512)	4.3 (1442)	4.1 (1703)	3.9 (1871)	4.2 (1879)
70–79	1.1 (366)	0.9 (314)	0.8 (296)	0.7 (262)	0.8 (277)	0.7 (224)	0.6 (256)	0.6 (301)	0.7 (296)
80 or greater	0.6 (192)	0.2 (78)	0.2 (68)	0.2 (63)	0.2 (72)	0.1 (33)	0.1 (52)	0.1 (58)	0.1 (55)
**Other procedures**									
All, *n*	4645	5087	4505	4330	4335	4401	6180	7118	6773
% Annual cases	4.2	4.1	3.3	3.1	3.1	3.6	4.0	4.1	4.2
BMI, % (*n*)									
Less than 40	31.2 (1447)	37.5 (1906)	36.2 (1630)	31.6 (1369)	29.4 (1274)	26.2 (1153)	22.7 (1405)	22.6 (1606)	18.2 (1233)
40–49	41.6 (1931)	37.6 (1915)	34.9 (1572)	35.2 (1526)	36.5 (1583)	39.1 (1719)	41.2 (2547)	40.7 (2895)	40.3 (2727)
50–59	19.1 (885)	18.2 (926)	20.1 (904)	23.7 (1025)	23.9 (1035)	24.7 (1088)	25.6 (1584)	26.4 (1881)	28.9 (1959)
60–69	5.8 (268)	5.4 (275)	7.0 (316)	7.5 (323)	7.6 (330)	7.7 (339)	7.8 (481)	7.7 (546)	9.3 (631)
70–79	1.7 (81)	1.1 (56)	1.4 (61)	1.6 (68)	2.3 (99)	1.8 (80)	2.2 (134)	2.1 (151)	2.6 (179)
80 or greater	0.7 (33)	0.2 (9)	0.5 (22)	0.4 (19)	0.3 (14)	0.5 (22)	0.5 (29)	0.5 (39)	0.6 (44)

**TABLE 2 |:** Bariatric surgery patient characteristics, MBSAQIP 2015–2023.

	All	BMI, kg/m^2^						*p*
		< 40.0	40.0–49.9	50.0–59.9	60.0–69.9	70.0–79.9	≥ 80.0	
All, % (*n*)	100.0 (1,262,454)	100.0 (321,652)	100.0 (644,759)	100.0 (228,902)	100.0 (53,464)	100.0 (10,612)	100.0 (3065)	
Age, mean (SD)	43.5 (11.8)	46.4 (11.5)	43.0 (11.8)	41.7 (11.6)	40.6 (10.9)	39.1 (9.9)	38.7 (10.0)	< 0.0001
Sex, % (*n*)								< 0.0001
Female	80.1 (1,011,282)	82.8 (266,390)	80.9 (521,769)	76.4 (174,932)	72.5 (38,761)	68.9 (7313)	69.1 (2117)	
Male	19.9 (251,172)	17.2 (55,262)	19.1 (122,990)	23.6 (53,970)	27.5 (14,703)	31.1 (3299)	30.9 (948)	
Race/ethnicity, % (*n*)								< 0.0001
White	57.8 (730,237)	59.6 (191,673)	58.1 (374,491)	56.3 (128,944)	53.2 (28,468)	48.9 (5192)	47.9 (1469)	
Black	18.6 (234,307)	13.3 (42,904)	18.5 (119,042)	23.3 (53,328)	27.4 (14,661)	31.5 (3342)	33.6 (1030)	
API	0.6 (7058)	0.8 (2704)	0.5 (3347)	0.4 (812)	0.3 (153)	0.4 (38)	0.1 (4)	
AIAN	0.4 (5432)	0.4 (1334)	0.4 (2628)	0.5 (1130)	0.5 (273)	0.6 (59)	0.3 (8)	
Hispanic	15.9 (200,360)	18.8 (60,312)	15.7 (101,036)	13.3 (30,535)	12.6 (6754)	12.6 (1340)	12.5 (383)	
Other/unknown	6.7 (85,060)	7.1 (22,725)	6.9 (44,215)	6.2 (14,153)	5.9 (3155)	6.0 (641)	5.6 (171)	
Primary procedure								< 0.0001
Sleeve gastrectomy	68.9 (870,445)	71.4 (229,777)	69.7 (449,158)	64.4 (147,408)	65.1 (34,828)	67.0 (7111)	70.6 (2163)	
RYGB	27.3 (344,635)	24.5 (78,852)	27.5 (177,186)	30.7 (70,207)	28.3 (15,127)	24.4 (2592)	21.9 (671)	
Other	3.8 (47,374)	4.0 (13,023)	2.9 (18,415)	4.9 (11,287)	6.6 (3509)	8.6 (909)	7.5 (231)	

Abbreviations: AIAN, American Indian or Alaska Native; API, Asian/Pacific Islander; RYGB, Roux-en-Y gastric bypass.

**TABLE 3 |:** Bariatric surgery patient comorbidities, MBSAQIP 2015–2023.

	All	BMI, kg/m^2^						*p*
		< 40.0	40.0–49.9	50.0–59.9	60.0–69.9	70.0–79.9	≥ 80.0	
All, % (*n*)	100.0 (1,262,454)	100.0 (321,652)	100.0 (644,759)	100.0 (228,902)	100.0 (53,464)	100.0 (10,612)	100.0 (3065)	
Diabetes status, % (*n*)								< 0.0001
Non-diabetic	74.7 (942,822)	72.3 (232,424)	76.7 (494,232)	73.4 (168,111)	71.6 (38,265)	71.3 (7567)	72.5 (2223)	
Diabetes, non-insulin	17.7 (223,678)	19.0 (60,985)	16.4 (105,582)	18.9 (43,360)	20.3 (10,868)	21.2 (2,254)	20.5 (629)	
Diabetes, insulin	7.6 (95,954)	8.8 (28,243)	7.0 (44,945)	7.6 (17,431)	8.1 (4,331)	7.5 (791)	6.9 (213)	
Comorbidities, % (*n*)								
Hypertension	45.5 (574,704)	46.9 (150,698)	42.4 (273,621)	49.5 (113,376)	54.7 (29,248)	57.1 (6064)	55.4 (1697)	< 0.0001
Sleep apnea	38.1 (480,514)	34.6 (111,247)	35.2 (226,740)	45.9 (105,162)	54.7 (29,219)	60.2 (6391)	57.3 (1755)	< 0.0001
COPD	1.4 (17,197)	1.1 (3618)	1.2 (7969)	1.8 (4020)	2.3 (1228)	2.8 (297)	2.1 (65)	< 0.0001
Smoker	7.5 (95,161)	6.4 (20,642)	7.6 (49,246)	8.4 (19,333)	8.9 (4756)	8.8 (933)	8.2 (251)	< 0.0001
Therapeutic anticoagulation	2.8 (35,364)	2.3 (7434)	2.6 (16,571)	3.5 (8050)	4.7 (2528)	5.5 (587)	6.3 (194)	<0.0001
Deep vein thrombosis	1.7 (21,037)	1.3 (4237)	1.6 (10,035)	2.1 (4819)	2.7 (1455)	3.5 (367)	4.0 (124)	<0.0001
Pulmonary embolism	1.3 (16,217)	0.9 (2978)	1.1 (7346)	1.8 (4013)	2.6 (1385)	3.5 (376)	3.9 (119)	<0.0001
Venous stasis	0.9 (10,861)	0.4 (1411)	0.7 (4527)	1.4 (3145)	2.4 (1281)	3.6 (379)	3.8 (118)	<0.0001
GERD	29.0 (366,124)	32.0 (103,078)	28.1 (181,415)	27.8 (63,710)	26.8 (14,306)	26.5 (2816)	26.1 (799)	<0.0001
Hyperlipidemia	23.0 (290,484)	28.7 (92,188)	21.5 (138,898)	20.5 (46,821)	19.3 (10,292)	16.9 (1796)	16.0 (489)	< 0.0001
Immunosuppressive therapy	2.0 (25,451)	2.2 (6935)	2.0 (12,719)	2.0 (4528)	1.9 (1021)	1.9 (198)	1.6 (50)	<0.0001
Percutaneous coronary intervention	1.6 (19,773)	1.8 (5894)	1.5 (9833)	1.4 (3286)	1.2 (639)	0.9 (93)	0.9 (28)	< 0.0001
Myocardial infarction	1.1 (14,144)	1.3 (4235)	1.1 (6932)	1.0 (2321)	1.0 (550)	0.8 (82)	0.8 (24)	< 0.0001
Previous cardiac surgery	0.9 (11,478)	1.1 (3513)	0.9 (5828)	0.8 (1744)	0.6 (334)	0.4 (44)	0.5 (15)	< 0.0001
Renal insufficiency	0.6 (7367)	0.5 (1659)	0.6 (3717)	0.7 (1539)	0.7 (361)	0.7 (69)	0.7 (22)	<0.0001
Dialysis	0.3 (4011)	0.3 (872)	0.4 (2268)	0.3 (726)	0.2 (121)	0.2 (19)	0.2 (5)	0.8207
Functional status, % (*n*)								<0.0001
Independent	99.2 (1,252,237)	99.5 (319,964)	99.4 (640,644)	98.9 (226,285)	97.8 (52,310)	95.8 (10,168)	93.5 (2,866)	
Partially dependent	0.6 (7120)	0.3 (976)	0.4 (2654)	0.9 (2017)	1.8 (954)	3.4 (365)	5.0 (154)	
Totally dependent	0.2 (2325)	0.2 (530)	0.2 (1104)	0.2 (450)	0.3 (143)	0.5 (57)	1.3 (41)	
Unknown	0.1 (772)	0.1 (182)	0.1 (357)	0.1 (150)	0.1 (57)	0.2 (22)	0.1 (4)	

*Note:* Grayed comorbidities indicate those that have a positive correlation with BMI.

Abbreviations: COPD, chronic obstructive pulmonary disease; GERD, gastroesophageal reflux disease.

**TABLE 4 |:** Thirty-day postoperative complications of MBS patients by BMI category, MBSAQIP 2015–2023.

	All	BMI, kg/m^2^						*p*
		< 40.0	40.0–49.9	50.0–59.9	60.0–69.9	70.0–79.9	≥ 80.0	
Postoperative transfusion	0.65 (8200)	0.74 (2404)	0.61 (3973)	0.61 (1395)	0.62 (334)	0.68 (72)	0.72 (22)	< 0.0001
Unplanned intubation	0.12 (1463)	0.09 (279)	0.10 (632)	0.15 (342)	0.25 (133)	0.49 (52)	0.82 (25)	< 0.0001
Postoperative ventilatory support (> 48 h)	0.07 (835)	0.04 (138)	0.05 (347)	0.09 (216)	0.15 (85)	0.28 (30)	0.62 (19)	< 0.0001
Unplanned ICU admission	0.59 (7475)	0.49 (1586)	0.52 (3328)	0.74 (1690)	1.15 (617)	1.64 (174)	2.61 (80)	< 0.0001
Readmission within 30 days	3.32 (41,938)	3.13 (10,080)	3.20 (20,595)	3.66 (8385)	4.12 (2202)	4.67 (496)	5.87 (180)	< 0.0001
Reoperation within 30 days	1.07 (13,533)	1.10 (3528)	1.03 (6655)	1.11 (2547)	1.19 (637)	1.20 (127)	1.27 (39)	0.0362
Reintervention within 30 days	1.0 (12,264)	0.96 (3075)	0.92 (5931)	1.07 (2452)	1.17 (627)	1.26 (134)	1.47 (45)	< 0.0001
MACE within 30 days	0.97 (975)	0.07 (235)	0.06 (413)	0.10 (231)	0.14 (75)	0.14 (15)	0.20 (6)	< 0.0001
Death within 30 days	0.08 (1029)	0.05 (145)	0.06 (409)	0.13 (305)	0.23 (122)	0.32 (34)	0.46 (14)	< 0.0001
Serious complication	0.85 (10,673)	0.68 (2186)	0.74 (4777)	1.07 (2452)	1.69 (906)	2.26 (240)	3.65 (112)	< 0.0001
Infectious complication	1.20 (15,136)	1.01 (3245)	1.13 (7275)	1.47 (3375)	1.80 (960)	1.99 (211)	2.28 (70)	< 0.0001

*Note:* Data represent percent and number, % (*n*).

Abbreviations: ICU, intensive care unit; MACE, major adverse cardiac events.

## Data Availability

The data that support the findings of this study are available on request from the corresponding author. The data are not publicly available due to privacy or ethical restrictions.

## Study Importance

- What is already known?
  - Extreme obesity (BMI ≥ 60 kg/m^2^) is the fastest-growing obesity subgroup in the National Health and Nutrition Examination Survey (NHANES), but prior studies frequently excluded these patients because of presumed data entry errors or confidentiality concerns.
  - Patients with extreme obesity remain poorly characterized, as most physicians have limited familiarity and hospitals/clinics are frequently ill-equipped to accommodate this degree of obesity.

- What does this study add?
  - Extreme obesity (BMI ≥ 60 kg/m^2^) is common (~5%) in patients undergoing metabolic and bariatric surgery (MBS), and increasingly higher BMI is associated with a greater disease burden for some but not all diseases.
  - While extreme obesity is associated with greater surgical risk, it remains relatively low and not risk prohibitive.

- How might these results change the direction of research or the focus of clinical practice?
  - Extreme obesity is common and requires multidisciplinary care, yet it has historically been over-looked in both clinical care and research.
  - Medically complex individuals with extreme obesity are being treated annually with MBS with relatively low risk, demonstrating that a high BMI should not rule out MBS as a therapeutic option.
  - Greater clinical and basic science investigation of obesity subtypes is needed to optimize treatment for extreme obesity.

## References

[1] Corpodean F, Kachmar M, Yang S, , “Association of Obesity Severity With Cardiometabolic and Renal Disease Burden in the United States,” Obesity 34 (2025): 323–328. 10.1002/oby.70099 PMC12834209 41250827

[2] Shen S, Brown KA, Green AK, and Iyengar NM, “Obesity and Cancer,” JAMA 335 (2026): 1341–1350. 10.1001/jama.2026.1114 41801209

[3] Kachmar M, Albaugh VL, Yang S, , “Disproportionate Increase in BMI of ≥60 kg/m^2^ in the USA,” Lancet Diabetes and Endocrinology 13 (2025): 463–465. 10.1016/S2213-8587(25)00069-5 40288378

[4] Münte E, Zhang X, Khurana A, and Hartmann P, “Prevalence of Extremely Severe Obesity and Metabolic Dysfunction Among US Children and Adolescents,” JAMA Network Open 8 (2025): e2521170. 10.1001/jamanetworkopen.2025.21170 PMC12268495 40668581

[5] Tumin D, Frech A, Lynch JL, Raman VT, Bhalla T, and Tobias JD, “Weight Gain Trajectory and Pain Interference in Young Adulthood: Evidence From a Longitudinal Birth Cohort Study,” Pain Medicine 21 (2020): 439–447. 10.1093/pm/pnz184 31386156

[6] Li W, Kelsey JL, Zhang Z, , “Small-Area Estimation and Prioritizing Communities for Obesity Control in Massachusetts,” American Journal of Public Health 99 (2011): 511–519. 10.2105/AJPH.2008.137364 PMC2642528 19150906

[7] Das SR, Kinsinger LS, Yancy WS, , “Obesity Prevalence Among Veterans at Veterans Affairs Medical Facilities,” American Journal of Preventive Medicine 28 (2005): 291–294. 10.1016/j.amepre.2004.12.007 15766618

[8] Koebnick C, Smith N, Huang K, Martinez MP, Clancy HA, and Kushi LH, “The Prevalence of Obesity and Obesity-Related Health Conditions in a Large, Multiethnic Cohort of Young Adults in California,” Annals of Epidemiology 22 (2012): 609–616. 10.1016/j.annepidem.2012.05.006 PMC3418481 22766471

[9] Hales M, Navarro M, Patel A, , “Barriers to Subspecialty Care Among Patients With Extremely Severe Obesity,” Annals of Internal Medicine 178 (2025): 1666–1667. 10.7326/ANNALS-25-01720 41021921

[10] Lagu T, Hannon NS, Rothberg MB, , “Access to Subspecialty Care for Patients With Mobility Impairment: A Survey,” Annals of Internal Medicine 158 (2013): 441–446. 10.7326/0003-4819-158-6-201303190-00003 23552258

[11] McGowan B, Ciudin A, Baker JL, , “A Systematic Review and Meta-Analysis of the Efficacy and Safety of Pharmacological Treatments for Obesity in Adults,” Nature Medicine 31 (2025): 3317–3329. 10.1038/s41591-025-03978-z PMC12532627 41039116

[12] Alsaqaaby MS, Cooney S, le Roux CW, and Pournaras DJ, “Sex, Race, and BMI in Clinical Trials of Medications for Obesity Over the Past Three Decades: A Systematic Review,” Lancet Diabetes and Endocrinology 12 (2024): 414–421. 10.1016/S2213-8587(24)00098-6 38723646

[13] Telem DA and Dimick JB, “Practical Guide to Surgical Data Sets: Metabolic and Bariatric Surgery Accreditation and Quality Program (MBSAQIP),” JAMA Surgery 153 (2018): 766. 10.1001/jamasurg.2018.0495 29617538

[14] American College of Surgeons, Optimal Resources for Metabolic and Bariatric Surgery (ACS, 2022).

[15] Aryee EK, Zhang S, Selvin E, and Fang M, “Prevalence of Obesity With and Without Confirmation of Excess Adiposity Among US Adults,” JAMA 333 (2025): 1726–1728. 10.1001/jama.2025.2704 PMC12006908 40244602

[16] Skinner AC, Perrin EM, Moss LA, and Skelton JA, “Cardiometabolic Risks and Severity of Obesity in Children and Young Adults,” New England Journal of Medicine 373 (2015): 1307–1317. 10.1056/NEJMoa1502821 26422721

[17] Littman AJ, Boyko EJ, McDonell MB, and Fihn SD, “Evaluation of a Weight Management Program for Veterans,” Preventing Chronic Disease 9 (2012): E99. 10.5888/pcd9.110267 PMC3437789 22595323

[18] Lawman HG, Ogden CL, Hassink S, Mallya G, Veur SV, and Foster GD, “Comparing Methods for Identifying Biologically Implausible Values in Height, Weight, and Body Mass Index Among Youth,” American Journal of Epidemiology 182 (2015): 359–365. 10.1093/aje/kwv057 PMC4528955 26182944

[19] Freedman DS, Lawman HG, Skinner AC, McGuire LC, Allison DB, and Ogden CL, “Validity of the WHO Cutoffs for Biologically Implausible Values of Weight, Height, and BMI in Children and Adolescents in NHANES From 1999 Through 2012 1, 2,” American Journal of Clinical Nutrition 102 (2015): 1000–1006. 10.3945/ajcn.115.115576 PMC4631693 26377160

[20] Müller MJ, Geisler C, Blundell J, , “The Case of GWAS of Obesity: Does Body Weight Control Play by the Rules?,” International Journal of Obesity 42 (2018): 1395–1405. 10.1038/s41366-018-0081-6 29795468

[21] Acosta A, Camilleri M, Dayyeh BA, , “Selection of Antiobesity Medications Based on Phenotypes Enhances Weight Loss: A Pragmatic Trial in an Obesity Clinic,” Obesity 29 (2021): 662–671. 10.1002/oby.23120 PMC8168710 33759389

[22] Yang C-H, Fagnocchi L, Apostle S, , “Independent Phenotypic Plasticity Axes Define Distinct Obesity Sub-Types,” Nature Metabolism 4 (2022): 1150–1165. 10.1038/s42255-022-00629-2 PMC9499872 36097183

[23] Sorokin EP, Cule M, Thanaj M, , “Genetic Subtypes of Type 2 Diabetes Are Distinguished Through the Lens of Abdominal MRI,” Frontiers in Genetics 16 (2025): 1605721. 10.3389/fgene.2025.1605721 PMC12307213 40741412

[24] Chami N, Wang Z, Svenstrup V, , “Genetic Subtyping of Obesity Reveals Biological Insights Into the Uncoupling of Adiposity From Its Cardiometabolic Comorbidities,” Nature Medicine 31, no. 11 (2025): 3801–3812. 10.1038/s41591-025-03931-0 PMC12618246 40940440

[25] Odoemelam CS, Naz A, Thanaj M, , “Identifying Four Obesity Axes Through Integrative Multiomics and Imaging Analysis,” Diabetes 74 (2025): 1168–1183. 10.2337/db24-1103 PMC12185968 40272846

[26] Mitsakos AT, Irish W, DeMaria EJ, Pories WJ, and Altieri MS, “Body Mass Index and Risk of Mortality in Patients Undergoing Bariatric Surgery,” Surgical Endoscopy 37 (2023): 1213–1221. 10.1007/s00464-022-09651-7 36156736

[27] Clapp B, Abi Mosleh K, Glasgow AE, , “Bariatric Surgery Is as Safe as Other Common Operations: An Analysis of the ACS-NSQIP,” Surgery for Obesity and Related Diseases 20, no. 6 (2024): 515–525. 10.1016/j.soard.2023.11.017 38182525

[28] Kitahara CM, Flint AJ, de Gonzalez AB, , “Association Between Class III Obesity (BMI of 40–59 kg/m2) and Mortality: A Pooled Analysis of 20 Prospective Studies,” PLoS Medicine 11 (2014): e1001673. 10.1371/journal.pmed.1001673 PMC4087039 25003901

[29] National Center for Health Statistics, 2021 NHANES Anthropometry Procedures Manual 2021 (CDC, 2021).

[30] Kachmar M, Garcia Navas HJ, Doiron JE, , “Preoperative Body Mass Index Reduction and 30-Day Outcomes After Metabolic and Bariatric Surgery: An MBSAQIP 2015–2023 Analysis,” Surgery for Obesity and Related Diseases 22 (2026): 980–988. 10.1016/j.soard.2026.04.005 42135179

[31] Rubino F, Cummings DE, Eckel RH, , “Definition and Diagnostic Criteria of Clinical Obesity,” Lancet Diabetes and Endocrinology 13 (2025): 221–262. 10.1016/S2213-8587(24)00316-4 PMC11870235 39824205

