# Supplementary material for: Extreme Obesity (BMI ≥ 60 kg/m2) Characterization up to and Beyond 80 kg/m2 in 1,262,454 Bariatric Surgery Patients
Source: Obesity (Silver Spring). Author manuscript; Available in PMC 2026 Sep 18. (PMC13588579; doi:10.1002/oby.70289)
Supplement: Supplementary file 1 [file NIHMS2206669-supplement-Supplementary_Information.docx]

**SUPPLEMENTAL MATERIALS**

**For**

**Extreme Obesity (BMI ≥60 kg/m^2^) Characterization Up to and Beyond 80 kg/m^2^ in 1,262,454 Bariatric Surgery Patients**

Vance L. Albaugh, MD, PhD^1,2,3*^, Hector Garcia Navas, MD^1,2^, Robert C. Ross, MD, MPH^1,2^, Florina Corpodean, MD^1,2^, Michael Kachmar, DO^1,2^, Denise M. Danos, PhD^4^, Steven B. Heymsfield, MD^1^, Peter Katzmarzyk, PhD^1^, Michael W. Cook, MD^2,5^, Philip R. Schauer, MD^1,2,3^

^1^Metamor Institute, Pennington Biomedical Research Center, Baton Rouge, LA

^2^Department of Surgery, Louisiana State University Health Sciences Center, New Orleans, LA

^3^Franciscan Missionaries of Our Lady Health, Baton Rouge, Louisiana

^4^Department of Behavioral & Community Health, Louisiana State University School of Public Health, New Orleans, Louisiana

^5^University Medical Center, New Orleans, LA

***Corresponding Author:**	Vance L. Albaugh, MD, PhD

Pennington Biomedical Research Center

6400 Perkins Rd

Baton Rouge, LA 70808, USA

vance.albaugh@pbrc.edu

**Supplemental Table S1: Perioperative outcome definitions.**

| **Perioperative Outcomes** | **Definition** |
| --- | --- |
| Infection Complications | Composite of the following: Septic shock, postoperative septic shock, postoperative organ space surgical site infection (SSI), postoperative pneumonia, postoperative deep incisional SSI, postoperative superficial incisional SSI, postoperative urinary tract infection (UTI). |
| Serious Complications | Composite of the following: Death, unplanned intubation, unplanned ICU admission, acute renal failure, progressive renal insufficiency, cardiac arrest requiring cardiopulmonary resuscitation (CPR), pulmonary embolism, myocardial infarction, postoperative UTI, postoperative sepsis, postoperative deep incisional SSI. |
| Major Adverse Cardiovascular Event (MACE) | Myocardial infarction, cerebrovascular accident (CVA), cardiac arrest requiring CPR. |
| Death | Death occurring within 30-day of index procedure. |
